# A peculiar case of primary central nervous system T-cell lymphoma with indolent behavior

**DOI:** 10.1007/s13760-022-01928-x

**Published:** 2022-04-02

**Authors:** David Wasilewski, Martin Janz, Arend Koch, Andreas Rosenwald, Ulrich Keller, Peter Vajkoczy, Katharina Faust, Ioannis Anagnostopoulos, Josefine Radke

**Affiliations:** 1 Department of Neurosurgery, Charité - Universitätsmedizin Berlin, corporate member of Freie Universität Berlin, Humboldt-Universität zu Berlin, Berlin Institute of Health, Charitéplatz 1, 10117 Berlin, Germany; 2 Department of Hematology, Oncology and Tumorimmunology, Charité - Universitätsmedizin Berlin, corporate member of Freie Universität Berlin, Humboldt-Universität zu Berlin, Berlin Institute of Health, Campus Benjamin Franklin, Hindenburgdamm 30, 12203 Berlin, Germany; 3 Max Delbrück Center for Molecular Medicine, Experimental and Clinical Research Center (ECRC), Lindenberger Weg 80, 13125 Berlin, Germany; 4 Department of Neuropathology, Charité - Universitätsmedizin Berlin, corporate member of Freie Universität Berlin, Humboldt-Universität zu Berlin, Berlin Institute of Health, Charitéplatz 1, 10117 Berlin, Germany; 5 Institute of Pathology and Reference Centre for Hematopathology, Universität Würzburg, Josef-Schneider-Straße 2, 97080 Würzburg, Germany; 6 Berlin Institute of Health (BIH), Anna-Louisa-Karsch-Str. 2, 10178 Berlin, Germany; 7 German Cancer Consortium (DKTK), Partner Site Berlin, Berlin, Germany; 8 Present Address: Institute of Pathology, University Medicine of Greifswald, Greifswald, Germany

## Introduction

Primary central nervous system lymphoma (PCNSL) is a rare extranodal non-Hodgkin’s lymphoma (NHL) confined to the CNS by the time of diagnosis. Primary central nervous system T-cell lymphoma (PCNSTCL) represents a very rare subtype accounting for approximately 2% of PCNSL cases [5, 6]. Frequencies described in the literature vary in different collectives and geographic locations ranging from 2% up to 17% [2, 3]. Given the rarity of PCNSTCL, insights into pathogenesis, clinical presentation and treatment modalities are limited and based on case series or case reports. Here, we present an unusual case of PCNSTCL in a treatment-naïve patient suggesting that some of these lymphomas pursue an indolent or atypical clinical course.

## Case presentation

**Fig. 1:**
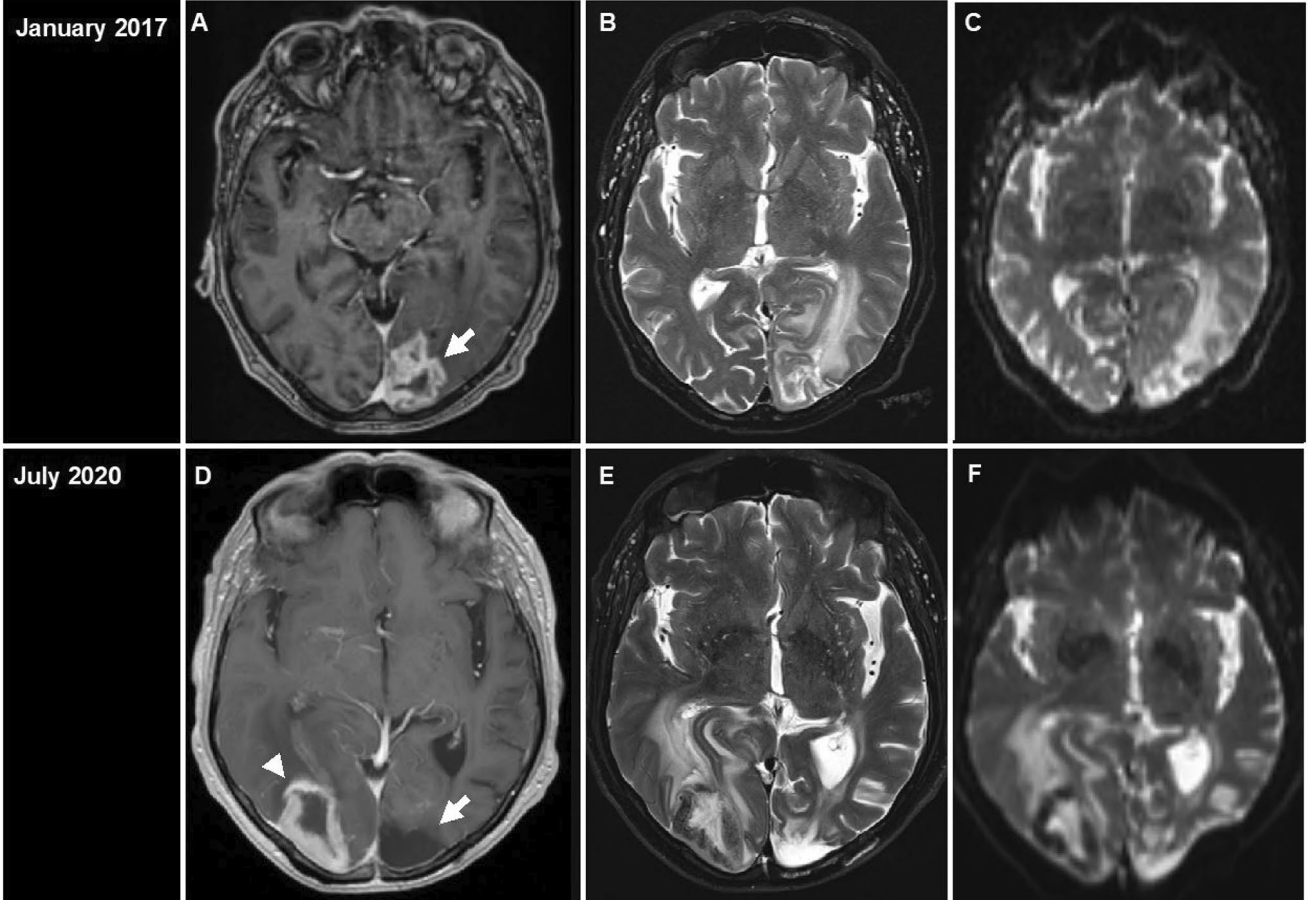
Post-gadolinium magnetic resonance imaging (MRI). T1 gadolinium-enhanced MRI from January 2017 (**A**–**C**) and July 2020 (**D**–**F**) showed abscess-like lesion with involvement of the left occipital lobe in January 2017 (**A**, white arrow) and right occipital lobe in 2020 (**D**, white arrowhead). Note the old, cyst-like cortical defect in the left occipital lobe in 2020 (**D**, white arrow), vasogenic edema and mass effect with displacement of the temporal horn of the right lateral ventricle

**Fig. 2:**
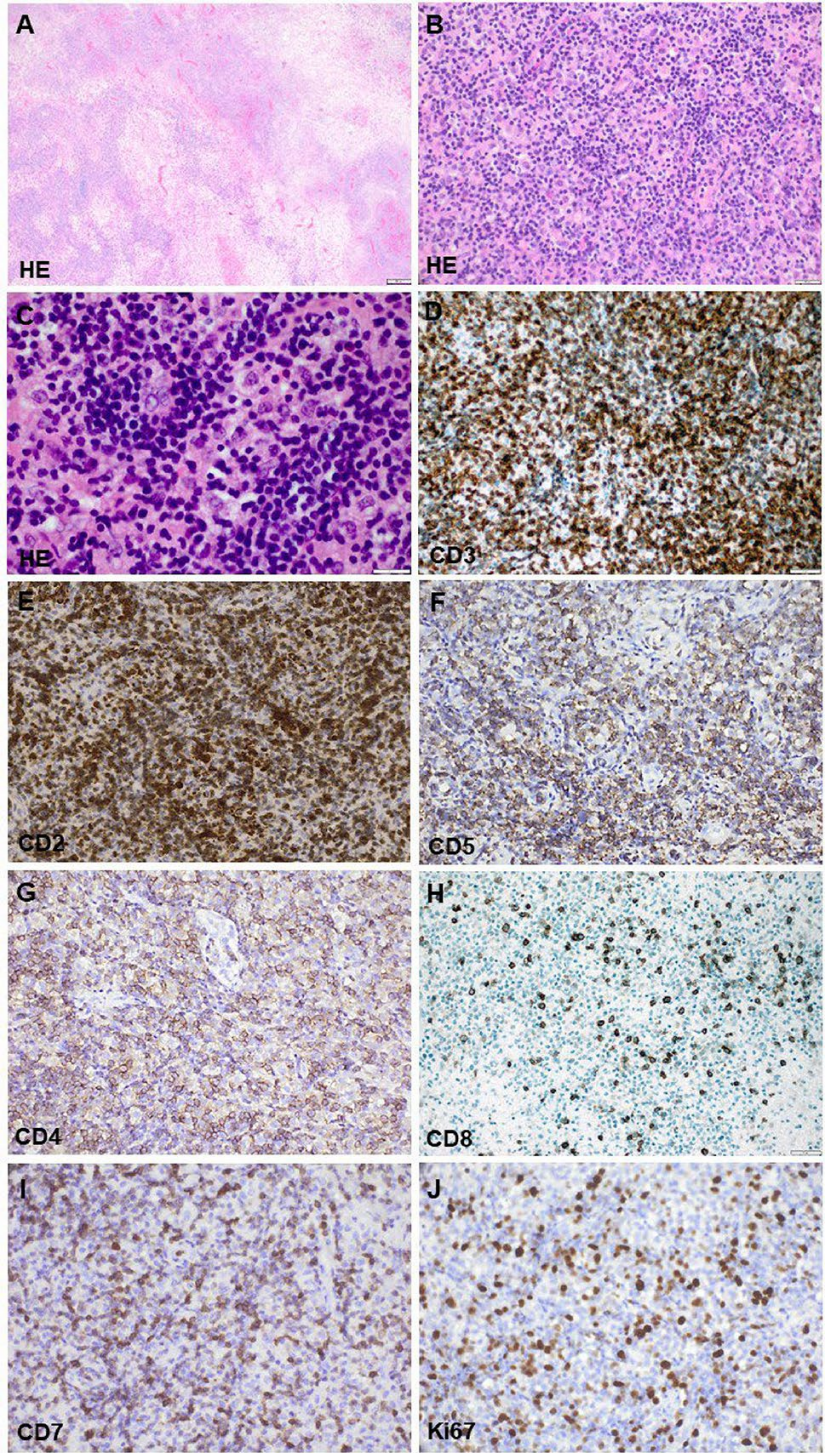
Histological evaluation of the lesion in 2020. The H&E staining revealed extensive areas of necrosis (**A**) with adjacent dense infiltrates composed of numerous small to medium-sized lymphoid cells (**B**, **C**). The lymphoid cells were identified to belong to the T-cell lineage and expressed CD3 (**D**), CD2 (**E**), CD5 (**F**) and CD4 (**G**) but not CD8 (**H**). Several of these T-cells demonstrated loss of CD7 expression (**I**). The growth fraction as detected by an antibody against Ki67 was up to 40% (**J**)

In July 2020, a 70-year-old man was submitted to our emergency department with a 1-week history of left-sided hemiparesis and homonymous hemianopia. Upon admission, the patient was fully alert and denied complaints such as headache, nausea, vomiting, or B symptoms. CRP (2.4 mg/mL; reference value < 5.0 mg/mL) and WBC count (10.33n/L) were normal, but he demonstrated a relevant lymphopenia (1.38/nL) and thrombocytopenia (72/nL). MRI revealed an intracerebral ring-enhancing lesion in the right occipital lobe with marked perifocal edema (Fig. 1). Prior history included evacuation of an abscess-like lesion in the left occipital lobe in January 2017 in our department. By that time, the histological evaluation revealed CNS tissue with dense T-cell dominated inflammatory infiltrates suggesting a necrotizing encephalitis. No conclusive results with respect to bacterial or viral pathogens were obtained in 2017. In fact, immunohistological stainings showed no expression of HIV-, HSV1-, HSV2-, CMV-, EBV-, JCV-, and Toxoplasma *gondii*-associated antigens (data not shown). Additional multiplex PCR analysis revealed no detection of CMV, EBV, HSV, VZV, or JCV. PAS and Grocott staining demonstrated no fungi and Gram staining showed no bacteria (data not shown). The patient was discharged without treatment. In July 2020, the patient presented a new abscess-like lesion in the contralateral (right) occipital lobe provoking a mass effect (Fig. 1). Intraoperatively, the lesion appeared purulent. Subsequently, intravenous broad-spectrum antibiotics including vancomycin and meropenem were initiated. All blood cultures yielded negative results, without evidence for bacterial or viral pathogens. HIV, SARS-CoV-2 testing as well as tuberculosis screening were negative. Interdisciplinary work-up excluded lymph node enlargement, pulmonary infiltrates, dental infections, or cancer metastases by whole body CT and abdominal MRI. Autoimmune testing showed no conclusive results. TEE excluded cardiac valve vegetation. Lumbar puncture was performed 11 days postoperatively and non-conclusive. Follow-up MRI 5 days after craniotomy and evacuation showed persisting edema with GTR. Antibiotic treatment was continued, and oral dexamethasone 3 × 8 mg/day administered due to edema and persisting neurological deficits, and down-tapered according to institutional SOPs. Histopathological analysis revealed a central necrosis with adjacent dense lymphocytic infiltrates (Fig. 2). Immunohistochemical stainings showed that the majority of lymphocytes were of T-cell origin expressing CD3, CD2, CD5, and CD4 (Fig. 2). Only rare CD20 and CD79a expressing B-cells were present. The lymphocytic infiltrate showed a Ki67-index of 40% (Fig. 2), which is why a lymphoma was considered as a differential diagnosis this time. The material was sent to a reference center for Hematopathology. Additional immunophenotyping revealed an aberrant T-cell immunophenotype with partial loss of CD7 expression (Fig. 2). The majority of T-cells expressed the beta (β) chain of the T-cell receptor (TCR). Only few T-cells (approx. 1%) expressed CD30 or PD1. There was neither evidence for a latent EBV-infection in the EBER *in-situ* hybridization, nor for expression of CD56, CD57, terminal deoxynucleotidyl transferase (TdT) or perforin. Further molecular analysis revealed presence of a monoclonal TCR gamma gene rearrangement. Re-evaluation of the previous lesion´s material (from 2017) revealed similar immunohistochemical results as well as evidence of a clonal expansion of T-cells after TCR gamma gene rearrangement analysis. The obtained amplificates from both biopsies (2017 and 2020) were different in size, a fact that has been described in occasional cases of T-cell lymphoma relapse [1]. Antibiotics were discontinued. Our tumor board proposed initiation of high-dose MTX after additional diagnostic work-up including bone marrow biopsy for detection of occult systemic lymphoma involvement, which proved to be negative. Diagnosis of a T-cell lymphoma not otherwise specified (NOS) was made and CNS-directed systemic therapy (high-dose MTX) following a modified PRIMAIN protocol was initiated [4]. From October 2020 to February 2021 six cycles were issued resulting in a complete remission. The patient is currently in follow-up, his condition has improved substantially (KPS 80%, month 5 post-chemotherapy).

## Discussion

We present an unusual case of a rather indolent PCNSTCL of the left occipital lobe in 2017, where the patient was discharged without definitive information on the underlying cause and without treatment. In 2020, an extensive interdisciplinary work-up led to the diagnosis of T-cell PCNSL, NOS. Disease recurrence without treatment years later indicates that T-cell lymphomas NOS might pursue a rather indolent clinical course. Patients with atypical clinical presentation and inconclusive histological findings should mandate early consultation of lymphoma experts to minimize the risk of missed opportunities for more timely diagnosis and treatment.

## Data Availability

All data generated during this study are included in this published article. Any further questions can be directed to the corresponding author.

## References

[1] Attygalle AD, Kyriakou C, Dupuis J, Grogg KL, Diss TC, Wotherspoon AC, Chuang SS, Cabecadas J, Isaacson PG, Du MQ, Gaulard P, Dogan A (2007). Histologic evolution of angioimmunoblastic T-cell lymphoma in consecutive biopsies: clinical correlation and insights into natural history and disease progression. Am J Surg Pathol.

[2] Choi JS, Nam DH, Ko YH, Seo JW, Choi YL, Suh YL, Ree HJ (2003). Primary central nervous system lymphoma in Korea: comparison of B- and T-cell lymphomas. Am J Surg Pathol.

[3] Ferreri AJ, Blay JY, Reni M, Pasini F, Spina M, Ambrosetti A, Calderoni A, Rossi A, Vavassori V, Conconi A, Devizzi L, Berger F, Ponzoni M, Borisch B, Tinguely M, Cerati M, Milani M, Orvieto E, Sanchez J, Chevreau C, Dell’Oro S, Zucca E, Cavalli F (2003). Prognostic scoring system for primary CNS lymphomas: the International Extranodal Lymphoma Study Group experience. J Clin Oncol.

[4] Fritsch K, Kasenda B, Schorb E, Hau P, Bloehdorn J, Mohle R, Low S, Binder M, Atta J, Keller U, Wolf HH, Krause SW, Hess G, Naumann R, Sasse S, Hirt C, Lamprecht M, Martens U, Morgner A, Panse J, Frickhofen N, Roth A, Hader C, Deckert M, Fricker H, Ihorst G, Finke J, Illerhaus G (2017). High-dose methotrexate-based immuno-chemotherapy for elderly primary CNS lymphoma patients (PRIMAIN study). Leukemia.

[5] Rubenstein JL, Gupta NK, Mannis GN, Lamarre AK, Treseler P (2013). How I treat CNS lymphomas. Blood.

[6] Shenkier TN, Blay JY, O’Neill BP, Poortmans P, Thiel E, Jahnke K, Abrey LE, Neuwelt E, Tsang R, Batchelor T, Harris N, Ferreri AJ, Ponzoni M, O’Brien P, Rubenstein J, Connors JM (2005). Primary CNS lymphoma of T-cell origin: a descriptive analysis from the international primary CNS lymphoma collaborative group. J Clin Oncol.

